# Contamination of fumonisin in feeds from different regions of China

**DOI:** 10.1186/s40104-026-01470-3

**Published:** 2026-07-24

**Authors:** Hua Sun, Dessalegn Lamesgen, Junjie Li, Juan Wang, Xuewu Li, Zhijie Yuan, Lijia Guo, Meng Liu, Fang Liu, Liwei Wang, Jianghong An, Jiangfeng He, Lvhui Sun

**Affiliations:** 1 Inner Mongolia Academy of Agriculture and Animal Husbandry Science, Hohhot, 010031 Inner Mongolia China; 2 State Key Laboratory of Agricultural Microbiology, Frontiers Science Center for Animal Breeding and Sustainable Production, College of Animal Science and Technology, Hubei Hongshan Laboratory, Huazhong Agricultural University, Wuhan, 430070 Hubei China; 3 Newhope Liuhe Co. Ltd., Beijing, 100102 China; 4 Hebei Panshuo Biotechnology Co., Ltd., Baoding, 071500 Hebei China

**Keywords:** China, Contamination, Co-contamination, Feeds, Fumonisin

## Abstract

**Background:**

Fumonisin (FUM) is a group of mycotoxins commonly found on corn, wheat, and their by-products, and has been linked to multiple animal and human health concerns. This study was conducted to investigate the contamination of FUM in feeds from different regions of China in 2025. A total of 1,128 feed samples, including 836 corn samples, 204 corn by-product samples, and 88 complete feed samples, were collected from different provinces of China for FUM analysis.

**Results:**

The analysis revealed a relatively high incidence of FUM contamination in the feed samples. Detection rates were 80.1%, 59.4%–100%, and 94.7%–100% in corn, corn by-products, and complete feeds, respectively, with median concentrations of 949 μg/kg, 2,825–74,040 μg/kg, and 1,050–1,396 μg/kg. Furthermore, corn samples collected from Henan, Jiangsu, and Shandong provinces exhibited relatively higher FUM concentration compared to other regions. Notably, 0.1% corn, 41.8% DDGS, 11.1% corn germ meal, 15.8% sprayed corn husk, 80.0% corn steep liquor, and 5.3% complete pig feed exceeded China’s safety standards for FUM. Moreover, the co-occurrence of FUM, AFB_1_, DON, and ZEN was quite common in both corn (39.5%–92.1%) and DDGS (73.1%–100%), with more than 90.0% of analyzed feed samples contaminated with two or more mycotoxins. Among them, the rates of over China’s safety standards for AFB_1_, DON and ZEN in corn were 7.9%, 1.3% and 2.6%, respectively, while did not in DDGS.

**Conclusion:**

This study reveals that the FUM was commonly present in feedstuffs and complete feeds of China in 2025. The results emphasize the significance of monitoring FUM levels in feeds and paying attention on co-contamination of mycotoxins, as well as implementing feed management and remedial strategies to reduce FUM exposure.

## Background

Mycotoxins are naturally occurring toxic secondary metabolites, mainly produced by filamentous fungi, including *Aspergillus*, *Alternaria*, *Claviceps*, *Fusarium*, and *Penicillium* [1, 2]. Currently, more than 500 mycotoxins have been identified in nature, which pose a potential threat to human and animal health [3]. Among these, aflatoxins (AFs), ochratoxins (OTs), fumonisins (FUMs), trichothecenes (TCT), zearalenone (ZEN), and patulin (PAT) are particularly critical due to their high prevalence, toxicity, and their role in continuous food safety issues worldwide [4]. The most predominant mycotoxins contaminated in grains, including corn, soybean, peanuts, wheat, barley, alfalfa, and their by-products, are aflatoxin B_1_ (AFB_1_), deoxynivalenol (DON), ZEN, and FUM [5–7]. Among these mycotoxins, AFB_1_, DON, and ZEN are common mycotoxins that may have carcinogenic, mutagenic, estrogenic, immunosuppressive, or gastrointestinal effects in animals [8, 9]. However, FUM contamination of feedstuffs poses a serious threat to feed and food safety worldwide in recent years [10, 11]. In particular, FUM is prevalent in feed ingredients, such as corn, distillers dried grains with solubles (DDGS), and other by-products, which account for a relatively high proportion of compound feeds and are also widely used in poultry, livestock, and aquaculture [12]. Therefore, contamination with FUM can easily trigger a health risk for animals, as well as cause substantial economic losses. The three main forms of FUM are fumonisin B_1_ (FB_1_), fumonisin B_2_ (FB_2_), and fumonisin B_3_ (FB_3_), where FB_1_ being the most prevalent (70%−80%) followed by FB_2_ and FB_3_, causing neurotoxicity, hepatotoxicity, nephrotoxicity, immunotoxicity, reproductive toxicity, and carcinogenicity in livestock and poultry [13–16].

On the one hand, the FUM can threaten the health of livestock and poultry by affecting feed quality and safety, on the other hand, the FUM in feed not only negatively impacts animal health but also poses risks to human health when converted into animal products [15, 16]. Thus, many countries have established safety standards for feed and feed ingredients. For example, America set maximum levels of FUM at 1,000–50,000 μg/kg for all kinds of feeds [17]. The European Commission set limitations of FUM at 5,000–60,000 μg/kg in feedstuffs and complete feeds [18, 19]. In 2017, General Administration of Quality Supervision, Inspection and Quarantine of the People’s Republic of China released the latest version of safety standards (GB 13078–2017) for FUM, which are 0–60,000 μg/kg, 5,000–50,000 μg/kg, 5,000–20,000 μg/kg, and 5,000–20,000 μg/kg, respectively, for corn and its by-products, concentrate supplement for ruminant, concentrated feed for pig and poultry, and complete feed for pig, rabbit, horse, poultry and fish (Table 1) [20].

**Table 1:** Limit standards of fumonisin in animal feeds in countries

Country	Feeds	Maximum limit, μg/kg
China	Corn and its by-products	60,000
	Concentrate supplement for calve and lamb	20,000
	Concentrate supplement for horse and rabbit	5,000
	Concentrate supplement for other ruminant	50,000
	Concentrated feed for pig	5,000
	Concentrated feed for poultry	20,000
	Complete feed for pig, rabbit and horse	5,000
	Complete feed for poultry	20,000
	Complete feed for fish	10,000
America	Feed for horse and rabbit	1,000
	Feed for pig and catfish	10,000
	Feed for breeding ruminant, breeding poultry, cow and layer	15,000
	Feed for edible ruminant	30,000
	Feed for edible poultry	50,000
	Feed for other animal species and pet	5,000
European union	Corn and its by-products	60,000
	Concentrate supplement and complete feed	5,000
	Feed for pig, horse, rabbit and pet	5,000
	Feed for fish	10,000
	Feed for poultry, calve and lamb	20,000
	Feed for adult ruminant	50,000

Today, global warming and climate change have increased the susceptibility of feeds such as corn and its by-products, soybeans, wheat, and complete feed to fungal colonization and FUM contamination [21, 22]. China, Brazil, Mexico, and Hungary, located in different regions, have successively reported contamination of corn and its products with FUM in recent years [23–26]. Thus, long-term monitoring of FUM levels in feed and feed ingredients is critical to keeping animal health and ensuring human food safety. China covers a large area spanning multiple climate zones, such as the northeast region, the Yangtze and the Yellow River basins, which are warm and humid with plenty of rainfall, increasing the crop's susceptibility to fungal infection [27–29]. Therefore, continuous monitoring of the levels of FUM in feed ingredients and complete feeds from these regions and other regions of China is of great significance to prevent livestock and poultry breeding exposure to FUM and to ensure feed and food safety. However, the contamination and distribution of FUM in feeds across different regions of China has not been sufficiently studied. Thus, the current study was conducted to investigate the individual and combined contamination status of FUM in nearly 1,100 feedstuffs and complete feeds collected from various regions of China in 2025. The study aims to provide a quantitative assessment of FUM occurrence and co-contamination patterns, offering a data support for feed safety management.

## Methods

### Sample collection and preparation

A total of 1,128 feed samples were collected from either feed companies or livestock farms in different regions of China in 2025. There were 1,040 feedstuff samples, including 836 corn, 110 DDGS, 20 corn gluten meal, 18 corn germ meal, 32 corn middling, 19 sprayed corn husk, and 5 corn steep liquor, as well as 88 complete feed samples, including 38 complete pig feed, 39 complete chicken feed, and 11 complete duck feed. According to the geographical regions of grain grown, these corn samples were primarily collected from the provinces of Anhui, Chongqing, Fujian, Guangdong, Guangxi, Guizhou, Hainan, Heilongjiang, Henan, Hubei, Hunan, Inner Mongolia, Jiangsu, Jiangxi, Jilin, Liaoning, Shaanxi, Shandong, Shanghai, Shanxi, Sichuan, and Yunnan. In total, 1,128 feed samples were analyzed for FUM. The collected feed samples were mixed and homogenized, and then stored in sealing bags at −20 °C before analysis.

### Extraction and quantification of FUM, AFB_1_, DON, and ZEN

The extraction and quantification of FUM, AFB_1_, DON, and ZEN from the feed samples collected from various regions of China in 2025 were performed as previously described [5, 6, 30]. A total of 1,128 feed samples were detected using commercially rapid test kits. Procedures of sample preparation and analyses were performed with commercially rapid test kits (Truthers Biotechnology Co., Ltd., Tianjin, China) according to their operating instructions. Briefly, 5 g of the mashed feed samples were mixed with a 25 mL of ethanol/distilled water solution (50/50, v/v), mixed at high speed for 3 min in a commercial mixer, and then centrifuged at 2,070 × *g* for 10 min, filtrated and collected the filtrate. Afterwards, a quantum dot fluorescence immunochromatographic assay were used for the rapid, sensitive, and quantitative detection of FUM (including FB_1_, FB_2_ and FB_3_), AFB_1_, DON, and ZEN. When mycotoxin is present in the samples, it could bind to the specific antibodies labeled with quantum dots and to inhibit the antibodies binding to the mycotoxin-BSA conjugate in the test line (T line), resulting in the fluorescence value of T line changing. Finally, the mycotoxin content is calculated based on the high and low fluorescence signal values of the T line. The limit of detection (LOD) was set at 200 μg/kg, 1 μg/kg, 100 μg/kg, and 10 μg/kg, respectively, for FUM, AFB_1_, DON, and ZEN in feed samples.

### Statistical analysis

All the data were analyzed using Microsoft Excel 2019 (Microsoft Corporation, Redmond, USA) and are expressed as means, median, maximum or percentages.

## Results

### Contamination of FUM in corn

A total of 836 corn samples were collected from 22 provinces in 2025 for FUM analysis (Table 2). Overall, the FUM was detected in 80.1% of corn in China, with the average level was 2,726 μg/kg. The median concentration and maximum level of FUM was 949 μg/kg and 200,000 μg/kg in corn, respectively. Among all the analyzed corn samples, only 1 corn sample (accounting for 0.1%) was contaminated with FUM at concentrations exceeding the maximum limit of 60,000 μg/kg set by China. In addition, according to the geographical regions of grain grown, all the corn samples source were divided as Heilongjiang (32), Jilin (47), Liaoning (76), Inner Mongolia (6), Shanxi (11), Anhui (37), Jiangsu (20), Jiangxi (26), Shandong (58), Shanghai (17), Fujian (11), Guangdong (131), Guangxi (17), Hainan (39), Henan (63), Hubei (26), Hunan (21), Shaanxi (49), Guizhou (6), Sichuan (95), Yunnan (6), and Chongqing (42) for comparative analysis. The FUM was detected in 29.4%−100% of corn from different provinces, with the average levels ranging from 637 to 6,954 μg/kg. Among these provinces, the top three median concentrations of FUM were 3,871 μg/kg, 2,500 μg/kg, and 2,067 μg/kg in corn from Henan, Jiangsu, and Shandong, respectively, and the top three maximum levels of FUM were 200,000 μg/kg, 29,040 μg/kg, and 18,803 μg/kg in corn from Shandong, Henan, and Guangdong, respectively. Notably, further analysis revealed that the 5^th^–95^th^ percentile ranges of FUM concentrations in Shandong were 1–12,717 μg/kg. These indicate that the maximum level of FUM (200,000 μg/kg) may be an extreme outlier, thereby causing the average level of FUM in Shandong to be skewed upward.

**Table 2:** FUM concentrations in corn^a^

Item	No. of samples	5^th^–95^th^ percentiles, μg/kg	Positive samples, μg/kg				Over standard rate, %
			**%**	**Mean**	**Median**	**Maximum**	
Heilongjiang	32	166–4,270	90.6	1,480	758	5,874	0
Jilin	47	106–9,054	89.4	2,311	901	15,378	0
Liaoning	76	246–4,590	96.1	1,911	1,475	9,513	0
Inner Mongolia	6	300–1,658	100	745	579	1,960	0
Shanxi	11	100–3,567	54.5	1,093	300	3,796	0
Anhui	37	0–8,501	62.2	2,988	825	12,600	0
Jiangsu	20	237–8,432	95.0	3,492	2,500	12,838	0
Jiangxi	26	0–7,377	38.5	1,848	147	7,400	0
Shandong	58	1–12,717	51.7	6,954	2,067	200,000	1.7
Shanghai	17	0–2,323	29.4	956	174	3,483	0
Fujian	11	728–1,903	100	1,183	926	2,131	0
Guangdong	131	114–4,162	92.4	1,517	871	18,803	0
Guangxi	17	182–1,747	82.4	637	300	3,670	0
Hainan	39	350–4,648	100	1,606	949	5,210	0
Henan	63	1,007–19,713	96.8	6,623	3,871	29,040	0
Hubei	26	100–3,667	92.3	1,615	665	4,757	0
Hunan	21	96–4,524	71.4	1,698	1,992	4,524	0
Shaanxi	49	242–13,768	95.9	2,700	863	16,000	0
Guizhou	6	268–3,398	83.3	1,777	1,510	3,470	0
Sichuan	95	7–2,816	53.7	745	299	6,450	0
Yunnan	6	100–1,948	83.3	1,145	1,367	1,996	0
Chongqing	42	0–10,660	83.3	4,111	1,773	11,330	0
Total	836	1–9,562	80.1	2,726	949	200,000	0.1

^a^Positive samples are defined as those with FUM ≥ 200 μg/kg (LOD)

### Contamination of FUM in corn by-products

A total of 204 samples of corn by-products, comprising 110 DDGS, 20 corn gluten meal, 18 corn germ meal, 32 corn middling, 19 sprayed corn husk, and 5 corn steep liquor, were collected in 2025 to analyze the level of FUM present in them (Table 3). The FUM was detected in 59.4%−100% of corn by-products, with the average levels ranging from 4,854 to 80,436 μg/kg. The highest median FUM concentration was 74,040 μg/kg in corn steep liquor. The maximum FUM level was 220,961 μg/kg in DDGS. Among all the analyzed corn by-product samples, 55 samples (27.0%) were contaminated with FUM at concentrations exceeding the maximal limit of 60,000 μg/kg set by China. Specifically, 46 DDGS (41.8%), 2 corn germ meal (11.1%), 3 sprayed corn husk (15.8%), and 4 corn steep liquor (80.0%), were contaminated with FUM at levels exceeding the Chinese safety standard concentrations.

**Table 3:** FUM concentrations in corn by-products^a^

Item	No. of samples	5^th^–95^th^ percentiles, μg/kg	Positive samples, μg/kg				Over standard rate, %
			**%**	**Mean**	**Median**	**Maximum**	
Distillers dried grains with solubles	110	640–139,468	97.3	54,199	39,910	220,961	41.8
Corn gluten meal	20	210–12,162	95.0	4,854	2,825	27,200	0
Corn germ meal	18	1,664–60,000	100	21,573	7,330	60,000	11.1
Corn middling	32	979–20,327	59.4	11,986	4,800	24,203	0
Sprayed corn husk	19	576–68,284	100	29,209	16,270	142,845	15.8
Corn steep liquor	5	33,106–129,140	100	80,436	74,040	137,239	80.0

^a^Positive samples are defined as those with FUM ≥ 200 μg/kg (LOD)

### Contamination of FUM in complete feeds

A total of 88 complete feed samples, including 38 complete pig feed, 39 complete chicken feed, and 11 complete duck feed, were collected in 2025 for analysis of FUM (Table 4). The FUM was detected in 94.7%−100% of complete feeds, with the average levels ranging from 1,508 to 2,493 μg/kg. The highest median concentration of FUM was 1,396 μg/kg in complete pig feed. The maximum FUM level was 18,030 μg/kg in complete chicken feed. Among all the analyzed complete feed samples, only 2 complete pig feed samples (5.3%) were contaminated with FUM at concentrations exceeding the maximal limit of 5,000 μg/kg set by China. Besides, the FUM concentrations in both complete chicken feed and complete duck feed were all within the safe standards in China.

**Table 4:** FUM concentrations in complete feeds^a^

Item	No. of samples	5^th^–95^th^ percentiles, μg/kg	Positive samples, μg/kg				Over standard rate, %
			**%**	**Mean**	**Median**	**Maximum**	
Complete pig feed	38	217–4,547	94.7	1,723	1,396	6,540	5.3
Complete chicken feed	39	245–9,762	94.9	2,493	1,210	18,030	0
Complete duck feed	11	340–3,855	100	1,508	1,050	4,600	0

^a^Positive samples are defined as those with FUM ≥ 200 μg/kg (LOD)

### Co-contamination of FUM, AFB_1_, DON, and ZEN in feeds

The co-contamination of FUM, AFB_1_, DON, and ZEN in feed samples in 2025 is presented in Table 5. A total of 102 feed samples, including 76 corn and 26 DDGS, were analyzed for co-contamination of mycotoxins. In addition to FUM, the AFB_1_, DON, and ZEN were detected at concentration of 0–440 μg/kg, 0–16,500 μg/kg, and 0–10,000 μg/kg in corn, respectively, as well as 0–50 μg/kg, 613–4,257 μg/kg, and 47–960 μg/kg in DDGS. Notably, the rates of over China’s safety standards for AFB_1_, DON and ZEN in corn were 7.9%, 1.3% and 2.6%, respectively, while did not in DDGS. Furthermore, more than 90.0% of analyzed feed samples showed co-occurrence rate of 39.5%−100% for FUM, AFB_1_, DON, and ZEN. More specifically, the co-contamination of FUM + AFB_1_, FUM + DON, FUM + ZEN, AFB_1_ + DON, AFB_1_ + ZEN, DON + ZEN, FUM + AFB_1_ + DON, FUM + AFB_1_ + ZEN, FUM + DON + ZEN, AFB_1_ + DON + ZEN, and FUM + AFB_1_ + DON + ZEN in corn were 40.8%, 92.1%, 84.2%, 43.4%, 42.1%, 88.2%, 40.8%, 39.5%, 82.9%, 42.1%, and 39.5%, respectively. Notably, the co-contamination of FUM + AFB_1_, FUM + DON, FUM + ZEN, AFB_1_ + DON, AFB_1_ + ZEN, DON + ZEN, FUM + AFB_1_ + DON, FUM + AFB_1_ + ZEN, FUM + DON + ZEN, AFB_1_ + DON + ZEN, and FUM + AFB_1_ + DON + ZEN in DDGS were 73.1%, 100%, 100%, 73.1%, 73.1%, 100%, 73.1%, 73.1%, 100%, 73.1%, and 73.1%, respectively.

**Table 5:** Co-contamination of FUM, AFB_1_, DON, and ZEN in feeds^a^

Item	Corn	Distillers dried grains with solubles
No. of samples	76	26
AFB_1_, μg/kg	0–440	0–50
DON, μg/kg	0–16,500	613–4,257
ZEN, μg/kg	0–10,000	47–960
FUM + AFB_1_, %	40.8	73.1
FUM + DON, %	92.1	100
FUM + ZEN, %	84.2	100
AFB_1_ + DON, %	43.4	73.1
AFB_1_ + ZEN, %	42.1	73.1
DON + ZEN, %	88.2	100
FUM + AFB_1_ + DON, %	40.8	73.1
FUM + AFB_1_ + ZEN, %	39.5	73.1
FUM + DON + ZEN, %	82.9	100
AFB_1_ + DON + ZEN, %	42.1	73.1
FUM + AFB_1_ + DON + ZEN, %	39.5	73.1

^a^FUM, fumonisin; AFB_1_, aflatoxin B_1_; DON, deoxynivalenol; ZEN, zearalenone; FUM + AFB_1_, feeds co-contaminated with FUM and AFB_1_; FUM + DON, feeds co-contaminated with FUM and DON; FUM + ZEN, feeds co-contaminated with FUM and ZEN; AFB_1_ + DON, feeds co-contaminated with AFB_1_ and DON; AFB_1_ + ZEN, feeds co-contaminated with AFB_1_ and ZEN; DON + ZEN, feeds co-contaminated with DON and ZEN; FUM + AFB_1_ + DON, feeds co-contaminated with FUM, AFB_1_ and DON; FUM + AFB_1_ + ZEN, feeds co-contaminated with FUM, AFB_1_ and ZEN; FUM + DON + ZEN, feeds co-contaminated with FUM, DON and ZEN; AFB_1_ + DON + ZEN, feeds co-contaminated with AFB_1_, DON and ZEN; FUM + AFB_1_ + DON + ZEN, feeds co-contaminated with FUM, AFB_1_, DON and ZEN; Positive samples are defined based on the following cutoff values: FUM ≥ 200 μg/kg (LOD), AFB_1_ ≥ 1 μg/kg (LOD), DON ≥ 100 μg/kg (LOD), ZEN ≥ 10 μg/kg (LOD)

## Discussion

With the rapid development of animal husbandry and breeding in China, the demand for feed resources is increasing [31]. As the primary energy source, corn accounts for approximately 60% of animal feed [32]. Meanwhile, due to the high protein content of DDGS and its economic competitiveness relative to soybean meal, its penetration into animal feed markets may increase [33, 34]. However, FUM contamination of corn and its by-products has become a growing problem in animal feed production [35]. Contamination of feed and food with FUM can endanger animal and human health and may reduce animal productivity, causing severe economic losses [36, 37]. Therefore, continuous monitoring of FUM levels in animal feed ingredients is crucial for keeping animal health and ensuring safety of human food products in the future. Given the widespread contamination, the present study investigated individual and combined contamination of FUM in feedstuffs and complete feeds collected from different regions of China in 2025. Overall, the analyzed FUM displayed a considerably high occurrence in the feed samples collected in 2025, with rates of 80.1% for corn, 59.4%–100% for corn by-products, and 94.7%–100% for complete feeds. Furthermore, considering the relatively high LOD of FUM, which may underestimate the risk of FUM’s actual exposure in feed and feed ingredients in China. These results indicated a widespread prevalence of FUM in feed ingredients and complete feeds from 22 provinces of China in 2025. However, a limitation of the present study is that individual FUM analogs (FB_1_, FB_2_, and FB_3_) were not quantified separately, which may limit the precision of toxicological interpretation. Future studies should prioritize the simultaneous quantification of FB_1_, FB_2_, and FB_3_ using high-resolution analytical techniques to better assess the toxicological risk associated with FUM contamination in feeds.

The average concentration of FUM (2,726 μg/kg) in corn determined in the present study was lower than previously reported concentrations from corn samples harvested in 2020 (3,811 μg/kg), while higher than 2021 (2,518 μg/kg) [30, 37]. The researchers adopted a similar method to detect FUM levels in 665 and 910 corn samples from 8–9 provinces in China, respectively [30, 37]. Notably, the FUM was most prevalent in Hainan, Fujian, Inner Mongolia, and Henan provinces, with positive rates of 100%, 100%, 100%, and 96.8%, respectively. Additionally, corn samples from Henan, Jiangsu, and Shandong provinces exhibited relatively higher FUM concentration compared to other regions, with the top three median concentrations were 3,871 μg/kg, 2,500 μg/kg, and 2,067 μg/kg, respectively. Geographically, these highly contaminated provinces are situated in the Jiangsu, Shandong, Henan and Anhui boundary region [38]. Henan, for instance, experiences an alternating temperate monsoon and subtropical climate, while Shandong and Jiangsu are heavily influenced by temperate monsoon patterns. Recent climate assessments indicate that these regions have been subjected to rising annual mean temperatures, more frequent extreme heat events, and fluctuating precipitation [39, 40]. Such climatic anomalies, specifically high temperatures coupled with intermittent severe rainfall or drought stress during the corn period, are highly conducive to the colonization of *Fusarium verticillioides* and the subsequent accumulation of FUM [41–43]. Furthermore, the warming climate in these regions have been shown to expand the occurrence areas and extend the active periods of agricultural insect pests [39, 40]. Consequently, the synergistic interplay of highly favorable climatic conditions for fungal growth and increased climate-driven pest pressure likely accounts for the elevated FUM contamination observed in Henan, Jiangsu, and Shandong provinces. Meanwhile, the current findings were basically consistent with previous studies in which higher FUM concentrations were detected in samples from Shandong, Anhui, Jiangsu, and Henan provinces than other regions of China [30, 44]. In addition to climate or presence of pests, harvest timing, storage, and transportation of feed ingredients are also worth considering, as they may significantly influence the formation and accumulation of FUM [45–47]. As the primary crop affected by FUM is corn, it is crucial to continuously monitor the levels of FUM in China, especially in different corn-producing regions, in the future.

The average levels of FUM in corn by-products ranged from 4,854 to 80,436 μg/kg. The 5^th^–95^th^ percentile ranges of FUM levels were 210–139,468 μg/kg in corn by-products in the current study, which was consistent with the previously reported results [30, 37, 44]. Meanwhile, the current findings emphasize the necessity of understanding the migration, transformation, and enrichment patterns of FUM during grain processing. The physicochemical properties of FUM, particularly its high-water solubility, mechanistically explain why toxin is further concentrated during wet milling [48], which accounts for the higher median FUM concentration found in corn steep liquor in this study. Conversely, starch is consumed while mycotoxins remain untouched during the dry milling and fermentation processes, effectively concentrating FUM and co-occurring mycotoxins up to several times in DDGS [48]. Furthermore, conventional analytical methods, such as the ELISA utilized in this study, may underestimate the true mycotoxin burden due to the formation of masked or matrix-bound FUM generated through thermal and chemical processing [49, 50]. Thus, number of studies have emphasized the necessity to measure modified FUM, particularly the O- and N-acylated forms of FUM [51, 52]. These masked forms frequently arise from plant defense mechanisms or are structurally modified during the processing of feed ingredients [52]. Because standard analytical protocols are often not optimized to detect these specific acylated modifications, the total FUM exposure in feedstuffs is systematically underestimated. While these masked forms evade detection, they can be hydrolyzed within the gastrointestinal tract of animals, releasing the toxic parent compounds and exacerbating systemic exposure [53]. In addition, it is worth noting that 27.0% of the analyzed corn by-products with FUM exceeded China’s safety standard, especially in corn steep liquor (80.0%), DDGS (41.8%), sprayed corn husk (15.8%), and corn germ meal (11.1%). However, due to the limited sample size of corn by-products, especially with only 5 samples of corn steep liquor, the results in this study possess a certain degree of uncertainty. These findings on corn steep liquor are preliminary and need to be validated in further studies with larger sample sizes. The findings from this study remind us that we need to be cognizant of the potential for contamination of corn by-products, including DDGS, corn gluten meal, corn germ meal, corn middling, sprayed corn husk, and corn steep liquor, which displayed a relatively high contamination of FUM with a median concentrations more than 2,800 μg/kg, as well as the samples contamination exceeding 59%. Therefore, in future, developing more advanced models to track the precise fate of mycotoxins from raw grains to finished by-products will provide critical and reliable scientific evidence required to optimize processing conditions and engineer targeted mitigation strategies.

The 5^th^–95^th^ percentile ranges of FUM occurrence were 217–9,762 μg/kg in the complete feed samples in the current study. Meanwhile, trends of FUM occurrence were similar in pig and chicken complete feeds (94.7%−94.9%), while duck complete feed exhibited more higher contamination (100%). This could be mainly attributed to the complete feeds being mixed with different feedstuffs and then containing FUM occurring in these commodities. Notably, 5.3% of all the analyzed complete feeds for pig contained FUM that exceeded the regulatory limits in China. Previous studies have shown that pigs fed FUM-contaminated feeds may cause widespread organ damage, such as pulmonary oedema, respiratory infections, as well as heart, liver, and kidney damage [54, 55]. Similarly, FUM exposure interfered with sphingolipid metabolism and immune signaling in poultry, and further increased diarrhea rate and mortality [55, 56]. Therefore, FUM can enter human food chain indirectly through carryover of toxins and their metabolites into animal tissues, meat, and eggs to increase potential risks for human health. Findings from this study indicated that pig, chicken, and duck complete feed showed high positive rates of FUM, and there was a risk of endangering the health of animals and people through the food chain.

Generally, the co-occurrence of two or more mycotoxins is likely to occur during natural mycotoxins contamination [5, 57, 58]. In addition to FUM, the AFB_1_, DON, and ZEN was detected in current research at concentration of 0–440 μg/kg, 0–16,500 μg/kg, and 0–10,000 μg/kg in corn, respectively, as well as 0–50 μg/kg, 613–4,257 μg/kg, and 47–960 μg/kg in DDGS. In contrast, China set limitations of AFB_1_, DON, and ZEN at 30–50 μg/kg, 0–5,000 μg/kg, and 500–1,500 μg/kg in corn and its products [6, 20]. Notably, the rates of over China’s safety standards for AFB_1_, DON and ZEN in corn were 7.9%, 1.3% and 2.6%, respectively, while did not in DDGS in this study. These findings revealed that the corn and DDGS of China in 2025 were commonly contaminated with FUM, AFB_1_, DON, and ZEN. Further analysis indicated that more than 90.0% of feed samples containing two or more mycotoxins. Notably, FUM + DON, FUM + ZEN, DON + ZEN, and FUM + DON + ZEN were more than 80% co-contaminated in corn. Meanwhile, the co-occurrence of two or more mycotoxins exceeded 70% in DDGS, especially the co-occurrence of FUM + DON, FUM + ZEN, DON + ZEN, and FUM + DON + ZEN were 100% within DDGS. These results were consistent with previous investigations that mycotoxins co-occurrence is a far more certain and prevalent issue in the feed and feed ingredients world widely [5, 6, 59–61]. The significance of mycotoxins co-contamination lies in the reality that different mycotoxins often exert additive or synergistic toxic effects on livestock and poultry, even when the concentration of each individual mycotoxin remains strictly below regulatory limits [10, 15, 62]. For instance, concurrent exposure to FUM and DON can synergistically impair the intestinal epithelial barrier and modulate immune responses, rendering animals highly susceptible to secondary enteric infections [63]. Similarly, the co-exposure of FUM and AFB_1_ has been reported to exacerbate hepatotoxicity and promote hepatocarcinogenesis more aggressively than either mycotoxin in isolation [64]. Currently, global feed safety regulations, including the safety standards in China, evaluate mycotoxins limits in strict isolation. However, this single-toxin pattern fundamentally underestimates the health risks associated with the combined toxicity of naturally co-occurring mycotoxins. Consequently, there is of great necessity to pivot toward a comprehensive, quantitative risk evaluation system that accounts for the synergistic impacts of concurrent mycotoxins exposure, which will be vital for refining feed regulatory thresholds and ensuring animal health.

## Conclusion

In conclusion, the survey of FUM contamination in 1,128 samples collected from different areas of China in 2025 showed that FUM was commonly present in feeds. Generally, the detection of FUM in corn, corn by-products, and complete feeds were 80.1%, 59.4%−100%, and 94.7%−100%, respectively. And corn samples collected from Henan, Jiangsu, and Shandong were relatively higher contaminated with FUM than other regions. Meanwhile, 0.1% corn, 27.0% corn by-products, and 5.3% complete pig feed exceeded China’s safety standards for FUM. Moreover, the co-occurrence of FUM, AFB_1_, DON, and ZEN were quite common in both corn (39.5%−92.1%) and DDGS (73.1%−100%), with more than 90.0% of analyzed samples contaminated with two or more mycotoxins. Notably, the rates of over China’s safety standards for AFB_1_, DON and ZEN in corn were 7.9%, 1.3% and 2.6%, respectively, while did not in DDGS. Based on this, the results remind us that the contamination of FUM in feeds of China in 2025 demonstrate a high incidence, therefore, it is necessary to strengthen the investigation and implement monitoring. Due to high co-occurrence rate of multiple mycotoxins in feeds, which may increase health risks of long-term feeding for animals, research focused on the combined toxicity of mycotoxins is particularly important. Meanwhile, effective strategies to prevent and control mycotoxins should aim to minimize mycotoxin contamination in feeds, ensure effective management, and reduce risks to animals.

## Data Availability

The datasets used and/or analyzed during the current study are publicly available.

## Abbreviations

AFB_1_: Aflatoxin B_1_
DON: Deoxynivalenol
FB_1_: Fumonisin B_1_
FB_2_: Fumonisin B_2_
FB_3_: Fumonisin B_3_
FUM: Fumonisin
ZEN: Zearalenone

## References

[1] Ismaiel A, Papenbrock J. Mycotoxins: producing fungi and mechanisms of phytotoxicity. Agriculture. 2015;5:1–46. 10.3390/agriculture5030492.

[2] Clarke LC, Sweeney T, Curley E, Duffy SK, Vigors S, Rajauria G, et al. Mycotoxin binder increases growth performance, nutrient digestibility and digestive health of finisher pigs offered wheat based diets grown under different agronomical conditions. Anim Feed Sci Tech. 2018;240:52–65. 10.1016/j.anifeedsci.2018.03.013.

[3] Aoyanagi MMCC, Budiño FEL, Raj J, Vasiljević M, Ali S, Ramalho LNZ, et al. Efficacy of two commercially available adsorbents to reduce the combined toxic effects of dietary aflatoxins, fumonisins, and zearalenone and their residues in the tissues of weaned pigs. Toxins (Basel). 2023;15(11):629. 10.3390/toxins15110629. 37999492 10.3390/toxins15110629 PMC10675588

[4] El-Sayed RA, Jebur AB, Kang W, El-Demerdash FM. An overview on the major mycotoxins in food products: characteristics, toxicity, and analysis. J Future Foods. 2022;2(2):91–102. 10.1016/j.jfutfo.2022.03.002.

[5] Zhao L, Zhang L, Xu Z, Liu X, Chen L, Dai J, et al. Occurrence of aflatoxin B1, deoxynivalenol and zearalenone in feeds in China during 2018–2020. J Anim Sci Biotechnol. 2021;12:74. 10.1186/s40104-021-00603-0. 34243805 10.1186/s40104-021-00603-0 PMC8272344

[6] Liu M, Xia Z, Zhang Y, Yang R, Luo W, Guo L, et al. Contamination of aflatoxin B_1_, deoxynivalenol and zearalenone in feeds in China from 2021 to 2024. J Anim Sci Biotechnol. 2025;16:66. 10.1186/s40104-025-01213-w. 40340773 10.1186/s40104-025-01213-w PMC12063307

[7] Marques SO, Deolindo GL, Brunetto ALR, da Veiga ALA, de Jesus RS, Da Gloria EM, et al. Combination of anti-mycotoxin additive in diet contaminated with multiple mycotoxins (aflatoxin, fumonisin, zearalenone and deoxynivalenol): effects on performance and health of lambs. Animals (Basel). 2025;15(19):2835. 10.3390/ani15192835. 41096430 10.3390/ani15192835 PMC12523754

[8] Feng Y, Lin Z, Wang H, Men Z, Shen J, Ma X. 3-Indolepropionic acid confers PXR-p53 binding to mitigate deoxynivalenol-induced hepatocyte apoptosis. Sci China Life Sci. 2025;68(9):2707–22. 10.1007/s11427-024-2911-8. 40553417 10.1007/s11427-024-2911-8

[9] Deng J, Yang JC, Feng Y, Xu ZJ, Kuča K, Liu M, et al. AP-1 and SP1 trans-activate the expression of hepatic CYP1A1 and CYP2A6 in the bioactivation of AFB_1_ in chicken. Sci China Life Sci. 2024;67(7):1468–78. 10.1007/s11427-023-2512-6. 38703348 10.1007/s11427-023-2512-6

[10] Xu R, Kiarie EG, Yiannikouris A, Sun L, Karrow NA. Nutritional impact of mycotoxins in food animal production and strategies for mitigation. J Anim Sci Biotechnol. 2022;13:69. 10.1186/s40104-022-00714-2. 35672806 10.1186/s40104-022-00714-2 PMC9175326

[11] Brown AA, Herrman T. Evaluating fumonisin contamination in cattle feed: impact on animal health, the agriculture industry and regulatory considerations. Curr Res Toxicol. 2025;8:100235–100235. 10.1016/j.crtox.2025.100235. 40469892 10.1016/j.crtox.2025.100235 PMC12133708

[12] Zheng L, Pender C, Gott P, Schwandt E, Ramirez S, Hofstetter U, et al. 136 mycotoxin contamination in United States corn and corn DDGS from 2019 and 2020 harvest. J Anim Sci. 2019;99:73–4. 10.1093/jas/skab054.121.

[13] Hou L, Yuan X, Le G, Lin Z, Gan F, Li H, et al. Fumonisin B_1_ induces nephrotoxicity via autophagy mediated by mTORC1 instead of mTORC2 in human renal tubule epithelial cells. Food Chem Toxicol. 2021;149:112037–112037. 10.1016/j.fct.2021.112037. 33548371 10.1016/j.fct.2021.112037

[14] Lumsangkul C, Tso KH, Fan YK, Chiang HI, Ju JC. Mycotoxin fumonisin B_1_ interferes sphingolipid metabolisms and neural tube closure during early embryogenesis in brown tsaiya ducks. Toxins (Basel). 2021;13(11):743–743. 10.3390/toxins13110743. 34822527 10.3390/toxins13110743 PMC8619080

[15] Voss KA, Plattner RD, Riley RT, Meredith FI, Norred WP. In vivo effects of fumonisin B_1_-producing and fumonisin B_1_-nonproducing *Fusarium moniliforme* isolates are similar: fumonisins B_2_ and B_3_ cause hepato- and nephrotoxicity in rats. Mycopathologia. 1998;141(1):45–58. 10.1023/A:1006810916344. 9725030 10.1023/a:1006810916344

[16] Chen J, Wen J, Tang Y, Shi J, Mu G, Yan R, et al. Research progress on fumonisin B_1_ contamination and toxicity: a review. Molecules. 2021;26(17):5238–5238. 10.3390/molecules26175238. 34500671 10.3390/molecules26175238 PMC8434385

[17] Center for Food Safety and Applied Nutrition and the Center for Veterinary Medicine at the Food and Drug Administration. Guidance for industry: fumonisin levels in human foods and animal feeds. 2001.

[18] European Commission. Directive 2002/32/EC of the European Parliament and of the Council of 7 May 2002 on undesirable substances in animal feed. Off J Eur Commun. 2002;10–22:16.

[19] European Commission. Commission recommendation of 17 August 2006 on the Presence of deoxynivelenol, zearalenone, ochratoxin A, T-2 and HT-2 and fumonisins in products intended for animal feeding (2006/576/EU). Off J Eur Commun. 2006:7–9.

[20] Ministry of Agriculture and Rural Affairs of the People’s Republic of China. Hygienical standard for feeds: GB 13078-2017. Beijing: Standards Press of China; 2017.

[21] Oke OE, Oni AI, Akosile OA, Oliyide KM, Ishola CA, Logunleko OA, et al. Heat stress and gut microbiome dynamics in poultry: interplay, consequences, and mitigation strategies. Anim Res One Health. 2025;1–20. 10.1002/aro2.70046.

[22] Perrone G, Ferrara M, Medina A, Pascale M, Magan N. Toxigenic fungi and mycotoxins in a climate change scenario: ecology, genomics, distribution, prediction and prevention of the risk. Microorganisms. 2020;8(10):1496. 10.3390/microorganisms8101496. 33003323 10.3390/microorganisms8101496 PMC7601308

[23] Wei L, Wang K, Liang X, Shi Y, Pan X, Wu X, et al. Occurrence and risk assessment of mycotoxins and their modified forms in maize from typical planting regions of China. Food Chem. 2025;483:144253. 10.1016/j.foodchem.2025.144253. 40233515 10.1016/j.foodchem.2025.144253

[24] de Matos NAV, Sartori AV, Soilo E, de Moraes MHP, Jacob SD. A survey on free and hidden fumonisins in Brazilian corn and corn-based products. Food Control. 2024;156. 10.1016/j.foodcont.2023.110135.

[25] Gilbert-Sandoval I, Wesseling S, Rietjens IMCM. Occurrence and probabilistic risk assessment of fumonisin B_1_, fumonisin B_2_ and deoxynivalenol in nixtamalized maize in Mexico city. Toxins (Basel). 2020;12(10):644–644. 10.3390/toxins12100644. 33036310 10.3390/toxins12100644 PMC7600745

[26] Zentai A, Szeitzné-Szabó M, Mihucz G, Szeli N, Szabó A, Kovács M. Occurrence and risk assessment of fumonisin B_1_ and B_2_ mycotoxins in maize-based food products in Hungary. Toxins (Basel). 2019;11(12):709. 10.3390/toxins11120709. 31817520 10.3390/toxins11120709 PMC6950155

[27] Xu F, Shi RJ, Liu LL, Li LJ, Wang HQ, Wang S, et al. The relationship of climate variables with the occurrence of fusarium pseudograminearum, an emerging pathogen causing fusarium head blight of wheat in Henan province of China. Plant Pathol. 2025;74(8):2306–18. 10.1111/ppa.70024.

[28] Cheng S, Feng X, Liu G, Zhao N, Liu J, Zhang Z, et al. Natural occurrence of mycotoxins in maize in north China. Toxins (Basel). 2022;14(8):521. 10.3390/toxins14080521. 36006182 10.3390/toxins14080521 PMC9414867

[29] Li R, Wang X, Zhou T, Yang D, Wang Q, Zhou Y. Occurrence of four mycotoxins in cereal and oil products in Yangtze Delta region of China and their food safety risks. Food Control. 2014;35(1):117–22. 10.1016/j.foodcont.2013.06.042.

[30] Li A, Hao W, Guan S, Wang J, An G. Mycotoxin contamination in feeds and feed materials in China in year 2020. Front Vet Sci. 2020;9:1016528. 10.3389/fvets.2022.1016528. 10.3389/fvets.2022.1016528 PMC9589091 36299638

[31] Deng ZC, Liu M, Cao KX, Khalil MM, Guan LL, Sun LH. Gut microbiome and postbiotics: bridging the dietary nutrition and feed efficiency in food-producing animals. Sci China Life Sci. 2025;68(12):3575–86. 10.1007/s11427-025-3063-5. 40974526 10.1007/s11427-025-3063-5

[32] Babatunde OO, Park CS, Adeola O. Nutritional potentials of atypical feed ingredients for broiler chickens and pigs. Animals (Basel). 2021;11(5):1196. 10.3390/ani11051196 10.3390/ani11051196 PMC8143358 33919422

[33] Brunetto ALR, Deolindo GL, dos Santos ALD, Nora L, de Vitt MG, de Jesus RS, et al. Performance, metabolism, and economic implications of replacing soybean meal with dried distillers grains with solubles in feedlot cattle diets. Fermentation (Basel). 2025;11(7):363. 10.3390/fermentation11070363.

[34] de Godoy MR, Bauer LL, Parsons CM, Fahey GC Jr. Select corn coproducts from the ethanol industry and their potential as ingredients in pet foods. J Anim Sci. 2009;87(1):189–99. 10.2527/jas.2007-0596. 18791159 10.2527/jas.2007-0596

[35] Pokoo-Aikins A, McDonough CM, Mitchell TR, Hawkins JA, Adams LF, Read QD, et al. Mycotoxin contamination and the nutritional content of corn targeted for animal feed. Poult Sci. 2024;103(12):104303. 10.1016/j.psj.2024.104303. 39299014 10.1016/j.psj.2024.104303 PMC11426393

[36] EFSA Panel on Contaminants in the Food Chain (CONTAM). Knutsen HK, Alexander J, Barregård L, Bignami M, Brüschweiler B, Ceccatelli S, et al. Risks for animal health related to the presence of fumonisins, their modified forms and hidden forms in feed. EFSA J. 2018;16(5):e05242. 10.2903/j.efsa.2018.5242. 10.2903/j.efsa.2018.5242 PMC7009563 32625894

[37] Hao W, Li A, Wang J, An G, Guan S. Mycotoxin contamination of feeds and raw materials in China in year 2021. Front Vet Sci. 2022;9:929904. 10.3389/fvets.2022.929904. 35847652 10.3389/fvets.2022.929904 PMC9281542

[38] Wang CL, Zhang SF, Zhang YS, Cui SS, Qin XF, Guenther A, et al. VOCs-driven ozone extremes during dry and wet heatwaves in the Jiangsu-Shandong-Henan-Anhui Boundary: integrating meteorological forcings and SHAP interpretation. Atmos Res. 2026;328:108396. 10.1016/j.atmosres.2025.108396.

[39] Wang T, Jiang Y, Chandio AA. Climate change, access to credit and grain productivity: evidence from counties in China’s major grain producing provinces. Front Sustain Food Syst. 2026;10:1800174. 10.3389/fsufs.2026.1800174.

[40] Zhang H, Tang Y, Chandio AA, Sargani GR, Ankrah TM. Measuring the effects of climate change on wheat production: evidence from Northern China. Int J Environ Res Public Health. 2022;19(19):12341. 10.3390/ijerph191912341. 36231641 10.3390/ijerph191912341 PMC9565046

[41] Czembor E, Stępień Ł, Waśkiewicz A. Effect of environmental factors on fusarium species and associated mycotoxins in maize grain grown in poland. PLoS ONE. 2015;10(7):e0133644. 10.1371/journal.pone.0133644. 26225823 10.1371/journal.pone.0133644 PMC4520617

[42] Li N, Zhao J, Zhang R, Deng L, Li J, Gao Y, et al. Effect of tebuconazole enantiomers and environmental factors on fumonisin accumulation and FUM gene expression in *Fusarium verticillioides*. J Agric Food Chem. 2018;66(50):13107–15. 10.1021/acs.jafc.8b04900. 30458614 10.1021/acs.jafc.8b04900

[43] Zhang J, Tang X, Cai Y, Zhou WW. Mycotoxin contamination status of cereals in China and potential microbial decontamination methods. Metabolites. 2023;13(4):551. 10.3390/metabo13040551. 37110209 10.3390/metabo13040551 PMC10143121

[44] Hao W, Guan S, Li A, Wang J, An G, Hofstetter U, et al. Mycotoxin occurrence in feeds and raw materials in China: a five-year investigation. Toxins (Basel). 2023;15(1):63. 10.3390/toxins15010063. 36668883 10.3390/toxins15010063 PMC9866187

[45] Tran TM, Ameye M, Phan LT, Devlieghere F, De Saeger S, Eeckhout M, et al. Post-harvest contamination of maize by *Fusarium verticillioides* and fumonisins linked to traditional harvest and post-harvest practices: a case study of small-holder farms in Vietnam. Int J Food Microbiol. 2021;339:109022. 10.1016/j.ijfoodmicro.2020.109022. 33340942 10.1016/j.ijfoodmicro.2020.109022

[46] Liu M, Zhao L, Gong G, Zhang L, Shi L, Dai J, et al. Invited review: remediation strategies for mycotoxin control in feed. J Anim Sci Biotechnol. 2022;13:19. 10.1186/s40104-021-00661-4. 35090579 10.1186/s40104-021-00661-4 PMC8796454

[47] Li T, Li J, Wang J, Xue KS, Su X, Qu H, et al. The occurrence and management of fumonisin contamination across the food production and supply chains. J Adv Res. 2024;60:13–26. 10.1016/j.jare.2023.08.001. 37544477 10.1016/j.jare.2023.08.001 PMC11156612

[48] Schaarschmidt S, Fauhl-Hassek C. The fate of mycotoxins during the primary food processing of maize. Food Control. 2021;121:107651. 10.1016/j.foodcont.2020.107651.

[49] Bryła M, Roszko M, Szymczyk K, Jędrzejczak R, Obiedziński M. Fumonisins and their masked forms in maize products. Food Control. 2015;59:619–27. 10.1016/j.foodcont.2015.06.032.

[50] Yang D, Ye Y, Sun J, Wang J, Huang C, Sun X. Occurrence, transformation, and toxicity of fumonisins and their covert products during food processing. Crit Rev Food Sci. 2022;64:3660–73. 10.1080/10408398.2022.2134290. 10.1080/10408398.2022.2134290 36239314

[51] Andrade P. Dietary risk assessment for fumonisins: challenges and prospects. Curr Opin Food Sci. 2023;541101080. 10.1016/j.cofs.2023.101080.

[52] Seiferlein M, Humpf H, Voss K, Sullards M, Allegood J, Wang E, et al. Hydrolyzed fumonisins HFB1 and HFB2 are acylated in vitro and in vivo by ceramide synthase to form cytotoxic N-acyl-metabolites. Mol Nutr Food Res. 2007;51(9):1120–30. 10.1002/mnfr.200700118. 17729221 10.1002/mnfr.200700118

[53] EFSA Panel on Contaminants in the Food Chain (CONTAM). Scientific opinion on the risks for human and animal health related to the presence of modified forms of certain mycotoxins in food and feed. EFSA J. 2014. 10.2903/j.efsa.2014.3916.

[54] Haschek WM, Gumprecht LA, Smith G, Tumbleson ME, Constable PD. Fumonisin toxicosis in swine: an overview of porcine pulmonary edema and current perspectives. Environ Health Persp. 2001;109(2):251–7. 10.1289/ehp.01109s2251. 10.1289/ehp.01109s2251 PMC1240673 11359693

[55] Anumudu CK, Ekwueme CT, Uhegwu CC, Ejileugha C, Augustine J, Okolo CA, et al. A review of the mycotoxin family of fumonisins, their biosynthesis, metabolism, methods of detection and effects on humans and animals. Int J Mol Sci. 2024;26(1):184. 10.3390/ijms26010184. 39796041 10.3390/ijms26010184 PMC11719890

[56] Ali MM, Delower H, Ridwan OA, Anum H, Shamsaldeen IS, Muhammad AM, et al. Integrating one health to mitigate the emergence and spread of antimicrobial resistance in livestock and aquaculture. Anim Res One Health. 2026;1–23. 10.1002/aro2.70064.

[57] Ma R, Zhang L, Liu M, Su YT, Xie WM, Zhang NY, et al. Individual and combined occurrence of mycotoxins in feed ingredients and complete feeds in China. Toxins (Basel). 2018;10(3):113. 10.3390/toxins10030113. 29518909 10.3390/toxins10030113 PMC5869401

[58] Deng J, Peng Z, Xia Z, Mo Y, Guo L, Wei J, et al. Five glutathione S-transferase isozymes played crucial role in the detoxification of aflatoxin B1 in chicken liver. J Anim Sci Biotechnol. 2025;16:54. 10.1186/s40104-025-01189-7. 40197593 10.1186/s40104-025-01189-7 PMC11977921

[59] Mngqawa P, Shephard GS, Green IR, Ngobeni SH, de Rijk TC, Katerere DR. Mycotoxin contamination of home-grown maize in rural northern South Africa (Limpopo and Mpumalanga Provinces). Food Addit Contam B. 2016;9(1):38–45. 10.1080/19393210.2015.1121928. 10.1080/19393210.2015.1121928 26600208

[60] Yalçin NF, Isik MK, Avci T, Oguz H, Yurduseven T. Investigation of mycotoxin residues in poultry feeds by LC MS/MS method. Ankara Univ Vet Fak. 2017;64(2):111–6. 10.1501/vetfak_0000002784.

[61] García-Díaz M, Gil-Serna J, Vázquez C, Botia MN, Patiño B. A comprehensive study on the occurrence of mycotoxins and their producing fungi during the maize production cycle in Spain. Microorganisms. 2020;8(1):141. 10.3390/microorganisms8010141. 31968531 10.3390/microorganisms8010141 PMC7023295

[62] Kulcsár S, Kövesi B, Balogh K, Zándoki E, Ancsin Z, Erdélyi M, et al. The co-occurrence of T-2 toxin, deoxynivalenol, and fumonisin B_1_ activated the glutathione redox system in the EU-limiting doses in laying hens. Toxins (Basel). 2023;15(5):305. 10.3390/toxins15050305. 37235340 10.3390/toxins15050305 PMC10222800

[63] Bracarense A, Lucioli J, Grenier B, Pacheco G, Moll W, Schatzmayr G, et al. Chronic ingestion of deoxynivalenol and fumonisin, alone or in interaction, induces morphological and immunological changes in the intestine of piglets. Brit J Nutr. 2012;107(12):1776–86. 10.1017/s0007114511004946. 21936967 10.1017/S0007114511004946

[64] Gelderblom W, Marasas W, Lebepe-Mazur S, Swanevelder S, Vessey C, Hall P. Interaction of fumonisin B_1_ and aflatoxin B_1_ in a short-term carcinogenesis model in rat liver. Toxicology. 2002;171(2–3):161–73. 10.1016/s0300-483x(01)00573-x. 11836022 10.1016/s0300-483x(01)00573-x

